# The Hyponatremic Hypertensive Syndrome in a Preterm Infant: A Case of Severe Hyponatremia with Neurological Sequels

**DOI:** 10.4061/2011/406515

**Published:** 2011-08-15

**Authors:** Vera van Tellingen, Marc R. Lilien, Jos F. M. Bruinenberg, Willem B. de Vries

**Affiliations:** ^1^Department of Neonatology, Wilhelmina Children's Hospital, University Medical Center Utrecht, P.O. Box 85090, 3508 AB Utrecht, The Netherlands; ^2^Department of Pediatric Nephrology, Wilhelmina Children's Hospital, University Medical Center Utrecht, P.O. Box 85090, 3508 AB Utrecht, The Netherlands; ^3^Department of Pediatrics, St. Elisabeth Hospital, P.O. Box 90151, 5000 LC Tilburg, The Netherlands

## Abstract

*Objective*. To report the irreversible severe neurological symptoms following the hyponatremic hypertensive syndrome (HHS) in an infant after umbilical arterial catheterization. *Design*. Case report with review of the literature. *Setting*. Neonatal intensive care unit at a tertiary care children's hospital. *Patient*. A three-week-old preterm infant. *Conclusions*. In evaluating a neonate with hyponatremia and hypertension, HHS should be considered, especially in case of umbilical arterial catheterization. In case of diagnostic delay, there is a risk of severe irreversible neurological damage.

## 1. Introduction

In the neonatal intensive care unit (NICU) population, hyponatremia is the most frequent encountered water and salt abnormality. With its broad differential diagnosis, it provides a challenge to the neonatologist. The most frequent causes are renal salt loosing through an immature kidney and the use of drugs such as diuretics [1]. Hypertension, however, is a relatively rare feature in children, especially in neonates, with an incidence in NICU's ranging from 0.7% to 3.2%, and renal arterial thrombosis following umbilical arterial catheterization as the leading cause [2, 3]. 

A rare clinical presentation of unilateral renal arterial stenosis is the hyponatremic hypertensive syndrome (HHS), characterized by activation of the renin angiotensin aldosterone (RAAS) system in the ischemic kidney, causing hypertension, and a counteracting effect on the other kidney, by different mechanisms leading to volume depletion and loss of electrolytes. This syndrome is caused by unilateral renal ischemia, due to stenosis or occlusion of a (branch of a) renal artery, and also occurs in a variety of other underlying disorders [4]. So far, only a few reports of HHS in children are available, with polydipsia, polyuria, enuresis, weight loss, volume depletion, and various neurological and behavioural symptoms as presenting symptoms [5].

We present a case of HHS in a preterm infant, with an extremely low sodium concentration, and discuss the difficulties encountered in treatment and the irreversible neurological sequels due to this potentially life-threatening metabolic disturbance.

## 2. Case Report

A preterm boy presented with extreme hyponatremia (plasma sodium of 101 mmol/L) at the 20th day after birth. He was born from a nulliparous woman at a gestational age of 31 weeks and 4 days after an uncomplicated pregnancy, followed by spontaneous rupture of membranes and antenatal corticosteroid administration. Apgar scores were 9 and 10 at 1 and 5 minutes, and the birth weight was 2080 grams. There were no complications during NICU stay over the first 3 days of life. An umbilical arterial catheter was inserted directly after birth, for the purpose of blood pressure monitoring, and removed after 3 days. Furthermore an umbilical venous catheter and subsequently a peripheral central venous catheter were inserted for the purpose of parenteral feeding. Routine cerebral ultrasonography showed an image consistent with the gestational age and mild periventricular flaring. No diuretics were administered.

At the third day of life the boy was transferred in good clinical condition to a regional hospital. He gained weight (from 1900 grams at the 3rd day to 2100 grams at two weeks after birth). At the age of 3 weeks rejection to feeding (until this moment consisting of 150 mL/kg/day breast milk with breast milk fortifier), weight loss (to a minimum of 1960 grams), irritability, hyperthermia, and polyuria were noticed. Cerebrospinal fluid analysis showed 219 leukocytes/mm^3^ with 12000 erythrocytes/mm^3^, after a traumatic lumbar puncture, thus a meningitis could no be excluded and intravenous antibiotics were started. Intravenous fluids, with a total volume of 150 mL/kg/day, containing 8 mg/kg/min glucose and 5 mmol/kg/day sodium, were administered in the regional hospital for 2 days (before return to the NICU).

The plasma sodium level had declined, from 140 mmol/L nine days before, to 101 mmol/L. There were no sodium levels examined in the interval between, but the level at the onset of symptoms was established at 112 mmol/L retrospectively in plasma stored at the laboratory of the regional hospital. The boy returned to the NICU under suspicion of a syndrome of inappropriate antidiuretic hormone secretion (SIADH) associated with the assumed meningitis, with initiated fluid restriction and sodium supplementation considered to be the appropriate therapy. The body weight at that time was 2030 grams.

We saw a pale, irritated neonate with tachypnea, arterial hypertension (104/60 mmHg, mean 78 mmHg), opisthotonus, and abnormal synchronized extensions of arms and legs. Additional to the plasma sodium level of 101 mmol/L, laboratory analysis revealed a mild hypokalemia, hypochloremia, and hypomagnesemia, with normal calcium and phosphate levels (for detailed information on all important laboratory results, see Table 1). Plasma osmolarity was 219 mOsmol/kg, and urea and creatinine levels were normal. Blood gas analysis showed a respiratory alkalosis with normal bicarbonate. Infection parameters were low, but liver enzymes and lactate were elevated (in the blood drawn several shortly after the seizure). Furthermore an elevated plasma B-type natriuretic peptide (BNP) of 1228 pmol/L was found (in children, there are no validated data on normal values available). Urinalysis showed no leucocytes, mild hematuria, low sodium and potassium, with proteinuria, glucosuria, and a urine osmolarity of 129 mOsmol/kg. Cultures of blood, urine and cerebrospinal fluid revealed no microorganisms.

The combination of hyponatremia and hypertension (defined as a mean blood pressure of >2 standard deviations for age and weight, in this case >75 mmHg) was suggestive of renal pathology. Abdominal Doppler ultrasound showed a right renal arterial thrombosis, partially calcified, and an oedematous appearance of the left kidney. It was suggested that the symptoms of this neonate resulted from an HHS secondary to a renal arterial thrombosis.

Blood pressure levels further increased in the first hours to a maximum of 108/62 (mean 96) mmHg. We chose to carefully normalise the blood pressure with intravenous dihydralazine, causing the right kidney to become completely afunctional (as demonstrated with 99 m technetium MAG3 renography in combination with the findings on Doppler ultrasound as mentioned before). Furthermore we supplemented sodium (by intravenous sodium chloride). Plasma sodium levels rose to, relatively fast within the first hours, above 120 mmol/L, and more slowly within the next 24 hours to normal. The dihydralazine and sodium supplementation could be gradually stopped after a few days, after which a normal blood pressure and electrolyte levels were maintained without any additional therapy, also urinalysis returned to normal.

Within a few minutes after return to the NICU the boy developed convulsions, successfully treated with phenobarbital. Cerebral ultrasound and magnetic resonance imaging (MRI) showed extensive white matter abnormalities and the presence of a sinus thrombosis of the superior sagittal, straight, and transverse sinus. Additional genetic screening revealed a mutation in the methylenetetra-hydrofolate reductase (MTHFR) gene.

Follow-up MRI at one and two months of age showed extension of the white matter abnormalities, with secondary haemorrhage, vacuolisation, and cyst formation. The gyration and myelinisation had increased, there were no signs of new ischemia, and all sinuses were recanalized. The child showed abnormal neurological behaviour (agitation, uncontrolled movements, and delayed motor development) at three months of followup. The parents of this child gave their informed consent to publication of this case report.

## 3. Discussion

In this case report, we describe a 3-week-old preterm boy, with extreme hyponatremia, hypertension and a dramatic neurological outcome, as a result of HHS following umbilical arterial catheterization. 

The typical combination of symptoms in HHS was first described in 1952 in adults [6], and the term HHS was established by Brown et al. in 1965 [4]. The majority of adult patients are elderly women with atherosclerosis [7]. In children, the syndrome is not encountered frequently and in neonates it is even more rare, with renal arterial stenosis following umbilical arterial catheterization being one of the described causes [8–10], accompanied by renal microthrombi in sepsis [11] and an association with Dexamethasone use [7]. The syndrome has been described more often in preterm than in term infants [8–11] and sometimes showed a lethal course [12, 13]. The high incidence of hyponatremia (30%) reported in hypertensive neonates, suggests that HHS is probably more common than we think [14].

**Figure 1:**
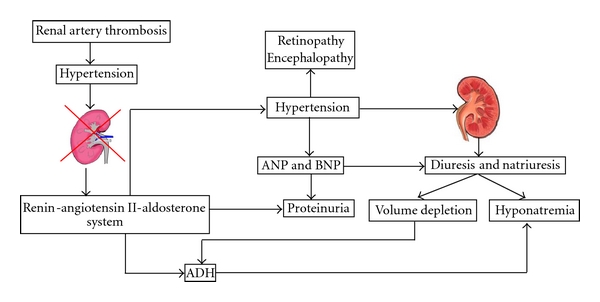
Suggested pathophysiology of the hyponatremic hypertensive syndrome, based on data from Nicholls 2006 [5].

HHS is thought to be due to a complex interplay of different mechanisms, with unilateral renal hypoperfusion and a counteracting effect of the contralateral normal kidney as major hallmarks (“two-kidney-one-clip hypertension”) [12] (Figure 1). The renal arterial thrombosis causes hypoperfusion of one kidney, which activates the RAAS system to cause hypertension. The contralateral nonstenotic kidney reacts to this hypertension by excreting water and sodium (pressure diuresis and natriuresis) [12, 15]. The hypertension additionally stimulates the cardiac atrial natriuretic peptide (ANP) and BNP to excrete more sodium and protein [16]. 

The resulting hypovolemia, probably together with an increased production of angiotensin II, stimulates antidiuretic hormone (ADH), further aggravating the hyponatremia. Furthermore, aldosterone causes renal potassium loss, which in turn stimulates renin secretion, causing a vicious circle [15]. Proteinuria, glucosuria, and hypercalciuria can also be present due to glomerular hyperfiltration in hypertension, increased renin activity, and probably even more extensive tubulointerstitial involvement [17].

In our case, the presence of an umbilical arterial catheter, and the finding of a mutation in the MTHFR gene (suggested to play role in homocysteine metabolism and enhancing atherosclerosis) were thought to be contributing factors in the development of the renal arterial thrombosis. At the moment of presentation at the NICU, our patient was thought to be already beyond the natriuretic phase of HHS (probably the severe hyponatremia finally resulted in sodium retention, explaining the low urine sodium) and most likely ADH had been turned on in reaction to the volume depletion. The initial sepsis-like presentation was retrospectively interpreted as the result of a combination of hypovolemia and central nervous system disturbances.

The prematurity was probably a major contributing factor leading to the severe outcome in this patient. This could have led to more extreme hyponatremia because preterms have a relatively low sodium intake, reduced tubular sodium reabsorption, and decreased glomerular filtration rate, impairing free water excretion [18]. Furthermore, recognizing the nonspecific clinical symptoms of HHS can be very difficult in a neonate. This diagnostic delay can result in long-lasting untreated hyponatremia and hypertension. 

The symptoms of HHS disappeared after normalising the blood pressure with a vasodilator agent (dihydralazine), causing the ischemic kidney to become nonvital by totally abrogating the blood flow, destroying the juxtaglomerular cells, and hence stopping renin production. There was a gradual decline in blood pressure in this patient, different from the potentially dangerous fall which can be seen with angiotensin-converting enzyme (ACE) inhibitors [19]. With severe volume depletion, cautious repletion is needed, which can probably also reduce arterial pressure by suppression of RAAS [20]. 

Correction of longstanding hyponatremia should be managed carefully, to minimise the risk of developing cerebral shrinking [21]. Final therapy of the underlying renal arterial stenosis was not necessary in this case, but can be achieved by balloon angioplasty, renal artery stenting, or uninephrectomy. However, in small children the first mentioned options can be technically impossible. 

The remarkable irreversible neurological features in this case are most likely to be the consecutive effect of a hypertensive and hyponatremic encephalopathy, aggravated by a diminished cerebral circulation due to hypovolemia and a sinus thrombosis. Furthermore the convulsions could also have led to irreversible damage to the vulnerable preterm brain. Previous case reports in older children mainly mention reversible neurological symptoms, and even reversible findings on computer tomography or MRI associated with HHS [19, 22–24] and only one infant dying from massive cerebral haemorrhage [13].

We realise that there are few limitations in the description of this case. First, there is a lack of (clinical and laboratory) information about the period before the patient represented with the severe hyponatremia. Unfortunately, no detailed information on water balance or 24-hour urine volumes during the period in which the patient developed the hyponatremia was available. As it is especially cumbersome to collect such data in newborns, this is very rarely done. Second, no data on plasma ADH, rennin, and aldosterone are available to confirm our suggested diagnosis.

This case was meant to describe the complex pathophysiology of HHS, and the possible misleading clinical features in a neonate. Furthermore we want to underline the risk of severe irreversible neurological damage when there is a diagnostic delay. We think that in evaluating a neonate with severe hyponatremia, HHS should be considered, especially if following umbilical arterial catheterization.

## Figures and Tables

**Table 1:** Laboratory values in plasma and urine, measured in the patient at readmission to the NICU.

Laboratory measurement	Measured value	Normal values
Plasma		
Sodium (mmol/L)	101	136–146
Potassium (mmol/L)	3.0	3.4–6.0
Chloride (mmol/L)	70	99–108
Magnesium (mmol/L)	0.66	0.70–1.00
Calcium, ionized (mmol/L)	1.25	0.95–1.50
Phosphate (mmol/L)	1.26	1.25–2.10
Glucose (mmol/L)	6.4	3.6–5.6
Osmolarity (mOsmol/kg)	219	280–295
Urea (mmol/L)	3.4	3.0–7.5
Creatinine (*μ*mol/L)	44	27–62
pH (arterial blood)	7.56	7.37–7.4
pCO_2_ (mmHg)	26	35–45
Bicarbonate (mmol/L)	22.9	22–28
Base excess (mmol/L)	1.0	−3.0–3.0
Lactate (mmol/L)	3.6	0.0–2.2
BNP (pmol/L)	1228	(Adult) <30–120
Hb (mmol/L)	8.4	(Adult) 5.9–8.4
Ht (mmol/L)	0.34	0.41–0.50
Erythrocytes (×10^12^/L)	3.94	3.20–4.80

Urine		
Sodium (mmol/L)	<10	Not available
Potassium (mmol/L)	19	Not available
Glucose (mmol/L)	16.1	Not available
Osmolarity (mOsmol/kg)	129	Not available
Protein (g/L)	>2.0	Not available
Creatinine (mmol/L)	1.5	Not available
pH	7.0	4.5–8.0
Erythrocytes (per *μ*L)	60	0–10
